# Squidpops: A Simple Tool to Crowdsource a Global Map of Marine Predation Intensity

**DOI:** 10.1371/journal.pone.0142994

**Published:** 2015-11-24

**Authors:** J. Emmett Duffy, Shelby L. Ziegler, Justin E. Campbell, Paige M. Bippus, Jonathan S. Lefcheck

**Affiliations:** 1 Tennenbaum Marine Observatories Network, Smithsonian Institution, Washington, DC, United States of America; 2 Virginia Institute of Marine Science, The College of William and Mary, Gloucester Point, Virginia, United States of America; 3 Smithsonian Marine Station, Fort Pierce, Florida, United States of America; 4 College of Charleston, Charleston, South Carolina, United States of America; Leibniz Center for Tropical Marine Ecology, GERMANY

## Abstract

We present a simple, standardized assay, the squidpop, for measuring the relative feeding intensity of generalist predators in aquatic systems. The assay consists of a 1.3-cm diameter disk of dried squid mantle tethered to a rod, which is either inserted in the sediment in soft-bottom habitats or secured to existing structure. Each replicate squidpop is scored as present or absent after 1 and 24 hours, and the data for analysis are proportions of replicate units consumed at each time. Tests in several habitats of the temperate southeastern USA (Virginia and North Carolina) and tropical Central America (Belize) confirmed the assay’s utility for measuring variation in predation intensity among habitats, among seasons, and along environmental gradients. In Belize, predation intensity varied strongly among habitats, with reef > seagrass = mangrove > unvegetated bare sand. Quantitative visual surveys confirmed that assayed feeding intensity increased with abundance and species richness of fishes across sites, with fish abundance and richness explaining up to 45% and 70% of the variation in bait loss respectively. In the southeastern USA, predation intensity varied seasonally, being highest during summer and declining in late autumn. Deployments in marsh habitats generally revealed a decline in mean predation intensity from fully marine to tidal freshwater sites. The simplicity, economy, and standardization of the squidpop assay should facilitate engagement of scientists and citizens alike, with the goal of constructing high-resolution maps of how top-down control varies through space and time in aquatic ecosystems, and addressing a broad array of long-standing hypotheses in macro- and community ecology.

## Introduction

The structure and functioning of ecosystems is largely dictated by the types and productivity of plants that populate them. These characteristics are in turn set by the supply of resources and the intensity of consumption by animals, commonly called bottom-up and top-down forcing, respectively. Bottom-up control is relatively well understood, with primary production following straightforward theoretical and empirical relationships with temperature and the availability of light, water, and mineral nutrients [1–3]. Because land plants are rooted and stationary, and phytoplankton tend to associate with water masses, vegetation can be mapped relatively easily, and as a result, remote sensing has yielded a picture of the distribution and biomass of primary producers at a level of detail unimaginable a few decades ago [4,5].

The geography of top-down control is much less well understood. While large-scale distributions of consumer biomass bear a strong imprint of bottom-up forcing (e.g. temperature, productivity, plant defenses, and elemental stoichiometry) [6–8], the mobility and diverse diets and feeding behaviors of animals have precluded a detailed global picture of top-down control equivalent to that available for primary production. Characterizing this variation in consumer pressure is especially important because numerous lines of evidence indicate that top-down control by predators tends to have stronger impacts and penetrates farther through the food web than does bottom-up control by resources [6]. Yet top predators, and large animals generally, are declining around the world, with often profound but poorly documented implications for ecosystem structure and functioning [7–9].

In short, there is a need for a systematic, empirical exploration of how feeding by herbivores and predators is distributed in space and varies through time [10]. Many important questions in both basic and applied ecology could be addressed with high-resolution spatial and temporal data on consumer activity [11]. Examples include: How does consumer pressure vary across the globe with latitude, temperature, resource supply, and along stress gradients [11–14]? To what extent are bottom-up and top-down control correlated with one another [15]? How accurately can top-down control be predicted by the biomass, body size distribution, and/or diversity of consumers [16,17]? How do management practices such as protected areas affect consumer pressure on native and introduced species [18]? What effect does extinction or decline of predators have on ecosystems [8]?

Mapping top-down control and answering such questions would be advanced by standardized methods that can be compared rigorously across ecosystem types and taxa. One promising approach to this goal involves exposure of standardized prey in different environments or conditions and comparing the rate of loss to predators [10]. Such experiments have been used widely by marine ecologists to study variation across habitats in rates of both herbivory [19,20] and predation [21–26]. Generally, these studies use living prey and most focus on estimating vulnerability of the particular prey taxon among habitats. But at larger geographic scales, such methods can be difficult because prey species differ among regions, prey are unavailable in some areas, and working with live prey poses logistical constraints. For many purposes, a standardized food type is desirable.

In this paper we present the design of a simple assay for measuring the relative feeding intensity of generalist predators in aquatic systems, and describe a suite of experiments that confirm its usefulness for estimating variation among habitats, seasons, consumer taxa, and along an environmental gradient (salinity). We show that feeding intensity measured with this assay correlates with the abundance and richness of predators, and present preliminary evidence that variation in feeding intensity across habitats is reflected in variation in standing biomass of prey. Importantly, the assay was designed with equal emphasis on simplicity, economy, and scientific rigor to ensure that it will be accessible to a wide range of users, including students and citizen scientists. Thus, this technique will facilitate the collection of locally and globally relevant scientific data at low cost.

## Methods

We developed and tested a simple, economical method for assaying relative feeding intensity by generalist aquatic consumers in a standardized and comparable way. We first describe the protocol that emerged from an iterative process of testing and modification, followed by results of deployments aimed at testing the assay’s usefulness for capturing variation in predation intensity among habitats, among seasons, and along an environmental gradient.

### The ‘squidpop’ assay of relative predation intensity

**Fig 1:**
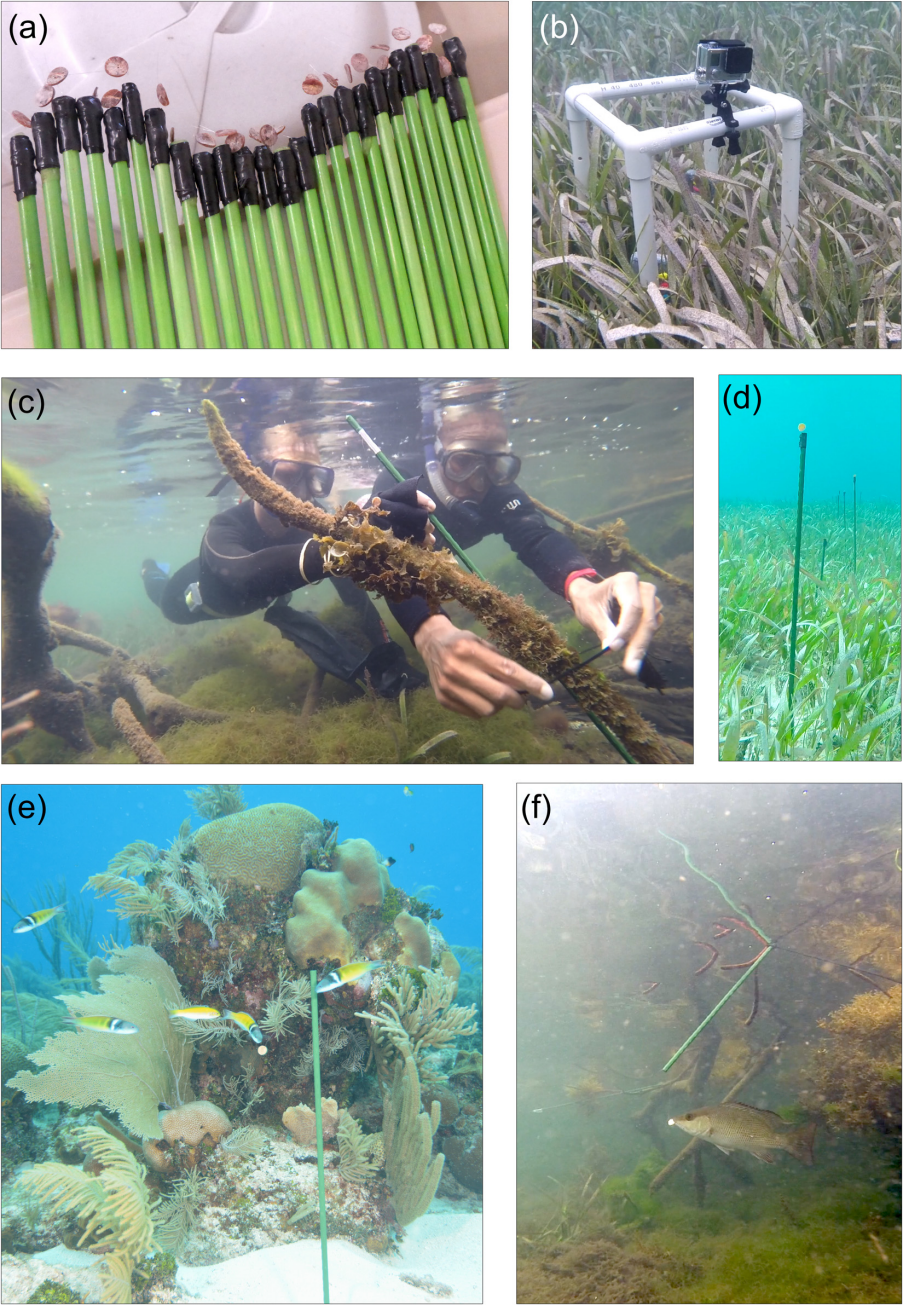
Design and deployment of the squidpop predation assay. (a) Assembled squidpops ready for deployment. (b) A GoPro camera deployed adjacent to a row of squidpops in seagrass to capture a record of predator visits. (c) Squidpops were attached to mangrove prop roots using cable-ties. (d) Squidpops deployed in a seagrass bed in Belize. (e) Bluehead wrasses (*Thalassoma bifasciatum*) attacking a squidpop in a reef habitat in Belize. (f) Gray snapper (*Lutjanus griseus*) attacking a squidpop in a mangrove habitat in Belize. The individuals pictured in Fig 1a) have given written informed consent (as outlined in PLOS consent form) to publish these case details.

We measured feeding activity of generalist marine carnivores, primarily fishes, using “squidpops”. The squidpop consists of a standard prey item: a small piece of dried squid mantle tissue, tethered to a fiberglass plant stake (0.6 x 60 cm) with light monofilament fishing line (Berkley^®^ Nanofil). Whole dried squid (Property Resources International Inc.) was purchased from a local Asian grocery store, but is also available through large online retailers (http://www.aliexpress.com/item/Free-shipping-giant-squid-350g-bag-imported-china-food-china-Specialties-Dried-food-Chinese-food/32307073752.html). The squidpop is assembled by first punching a 1.3-cm diameter disc from the squid mantle using a cork borer. A length of monofilament line is threaded through the squid disc and tied to it securely, then the other end of the line is attached to the end of the plant stake with electrical tape, leaving 1–2 cm of line with the squid piece on the end (Fig 1a). The short length of the line helps prevent tangling the tethers when squidpops are bundled together.

After assembly, the array of 25 squidpops is bundled together and transported to the field. Squidpops are deployed by pushing the stakes securely into the substratum (Fig 1d). One assay involves deployment of 25 replicate squidpops, which are thrust into the sediment roughly along a depth contour, separated by 1–2 m, depending on visibility and the nature of the terrain. The stakes are typically arranged in one or two rows, but the spatial arrangement is of secondary importance to ensuring that the squidpops can be reliably relocated and retrieved at the end of the deployment. For this reason it is helpful to mark the beginning and end of the array, e.g., with PVC poles, surveyor’s flags, or other markers. Where deployment in sediment is not practical, the stakes can be secured to other objects (dead coral or other non-living substratum, mangrove prop roots, etc.) using cable ties (Fig 1c).

We scored squidpops for the presence or absence of the squid bait one hour after deployment, and then again after 24 hours. Scoring at both time periods is important in capturing relative rates of bait consumption across the large range in predation intensity across habitats and regions (see Results). At each time period, the squid is scored as either present (score = 1) if any part of the squid remains, or absent (score = 0) if it is completely gone. Partial losses are not recognized as they are rare, subjective and time-consuming to score, and in our experience, scoring partial losses produces results nearly identical to those from binary scoring. Specifically, scoring after one hour is useful in many tropical habitats where predation is generally intense (and all baits are typically consumed after 24 hours, yielding no useful data on comparative predation intensity). Scoring after 24 hours captures data in regions such as high latitudes, where predation intensity is often low and scoring after one hour reveals little or no variation in bait loss.

The final design of the squidpop assay reflects an extended process of experimentation and modification. Dried squid was chosen as the standard food because it is of marine origin, tough enough that it is rarely if ever dislodged by processes other than predation, resistant to degradation over 24 hours, palatable to a wide range of fishes and invertebrate predators, and is available online in dry form that can be stored and shipped without refrigeration. To present the bait, we use fiberglass stakes rather than ropes [27] or clothespins [19,28] because stakes can be anchored in the widest range of marine bottoms, including soft sediments, oyster bars, and reef habitats. Where they cannot be pushed securely into the bottom, they can be affixed to other objects using cable-ties or other fastener. The stakes used are light green fiberglass plant stakes, ~60 cm (2 feet) in length, and available in packs of 20 from Amazon.com (EcoStake, http://amzn.to/1Lx6EDX). The stakes are corrosion-proof, inexpensive, and lightweight but sturdy, allowing them to be pushed into most types of sediments and gravel (Fig 1) and reused repeatedly. The light green color allows them to be relocated underwater in most habitats with moderate visibility and is superior to darker green stakes in this respect. It is likely that the presentation of the squid bait, including stake color and length, influence predator feeding rates, emphasizing the importance of adhering as closely as possible to the specifications of the assay to ensure rigorous comparability in results across space and time.

### Protocol development

Initial tests of the squidpop protocol used transparent acrylic rods (60 x 0.25 cm) or hollow metal plant stakes, and longer monofilament tethers (~10 cm). To determine whether these protocol variations influenced measured squid loss we conducted two experiments. First, we tethered squid discs to transparent acrylic rods and dark green metal plant stakes (N = 20 each) and deployed them in a paired arrangement along a 100-m transect in a seagrass meadow in the York River estuary, Virginia, USA (37.254, -76.447). At points along the transect a green metal squidpop was deployed 1 m to one side, and a transparent acrylic squidpop was placed 1 m to the other side of the transect, with the side assigned to each type alternating along the transect. Squidpops were scored after one hour and after 24 hours as described above.

The second experiment tested whether length of the monofilament tether influenced measured predation rates. In this case squid discs were tethered to green fiberglass stakes with a monofilament tether of either ~1 cm or ~10 cm in length (N = 25 each) and deployed in two parallel rows in a reef area near the Smithsonian Tropical Research Institute’s Bocas del Toro field station in Caribbean Panama (9.3519, -82.258). Squidpops were scored after one hour and after 24 hours as described above.

Using the final iteration of the protocol described in the previous section, we conducted several sets of squidpop assays to evaluate the effectiveness of the method for capturing variation in predation intensity across seasons, habitats, and environmental gradients.

### Comparisons of predation intensity and predator abundance across habitats

To quantify differences in predation intensity among habitats and their relationship to predator abundance and diversity, we deployed a total of 19 squidpop assays in coral reef (6 deployments), seagrass (5), mangrove (4), and bare sand (4) habitats in the vicinity of the Smithsonian Institution’s field station at Carrie Bow Cay, on the Belize Barrier Reef (16.803, -88.082). Assays were conducted in Sep-Oct 2014 and Feb 2015 as described above, and each assay consisted of 25 individual squidpop units deployed along one or two parallel rows. Squidpop stakes were thrust into the sediment in reef, seagrass (primarily *Thalassia testudinum*), and open sand habitats, and cable-tied to prop roots in the mangrove habitat. Squidpops were scored in situ by a diver or snorkeler after one hour as either present (1) or absent (0), and then again after 24 hours, after which all stakes were retrieved. For most deployments, a GoPro camera was positioned 1–3 meters away from the row of squidpops (Fig 1b) and recorded video for the first hour of deployment to give a sense of the identity and abundance of predators visiting and/or interacting with the squidpops.

To evaluate whether spatial patterns in predation intensity reflected differences in predator abundance and/or diversity among habitats, divers conducted quantitative visual surveys of fishes during squidpop deployments in each habitat following methods modified from Lewis [29] and Rotjan [30]. In brief, fish species abundance and size were quantified along a 25 m x 4 m belt transect deployed in close proximity to the location of the assays. Two passes along each transect were conducted to ensure that the visual surveys were comprehensive, and captured fishes of varying size classes. During the initial pass, a solitary diver slowly swam along the transect (~5 m/min) and recorded the size and abundance of large fishes (>20 cm total length) within a moving 4 m-wide band of the substrate (oriented perpendicular to the transect). On the second pass along the same transect, the diver recorded size and abundance of smaller fishes (≤ 20cm total length). All fishes were identified to species, and total length was estimated to the nearest 5 cm.

### Tests of variation in predation across seasons and latitude

To assess variation in predation intensity across sites and seasons we deployed squidpops monthly between April and November 2014 in seagrass (primarily *Zostera marina*) habitats at Goodwin Island (37.224, -76.392), in the York River estuary, Virginia, and at Hoop Hole Creek (34.426, -76.451), North Carolina, USA. At each deployment, squidpops were placed at depths of roughly 0.5–1.0 m MLW along a 50 m transect, marked every 3 m by surveyor’s flags to aid relocation of squidpops in the often turbid water. One squidpop was thrust into the sediment on either side of the flag about 1 m from the transect so that the squid bait was 15–25 cm above the sediment. Squidpops were scored in situ by a snorkeler after one hour as either present (1) or absent (0), and then again at 24 hours, after which stakes were retrieved. We recorded salinity and temperature during deployment at each site.

Turbid water precluded accurate diver surveys of fish assemblages at the seagrass sites in Virginia and North Carolina. Instead we used these deployments to search for correlations across sites and seasons between feeding intensity estimated with squidpops and abundance of potential prey. To characterize the prey community associated with seagrass at the two sites, we collected ten samples of epifaunal invertebrates associated with seagrass from each site during each month of deployment. Samples of seagrass blades with associated epifauna were enclosed in a 250 μm-mesh drawstring bag underwater, then the drawstring was pulled tight, and the enclosed seagrass was cut at the level of the bag closure. In the laboratory the mobile epifaunal invertebrates were separated from the seagrass, preserved in 70% ethanol, and identified to the lowest taxonomic level, usually species. Biomasses of epifaunal invertebrates were estimated by washing the preserved animals through a set of nested sieves and calculating the biomass retained on each sieve using empirical equations derived by Edgar [31].

### Tests of variation in predation along an environmental (salinity) gradient

To assess variation in predation intensity along a major environmental gradient, we deployed squidpop assays monthly between June and October 2014 in salt marsh habitats at five sites spanning the head to the mouth of the York River, Virginia, USA, and out to an embayment of the Atlantic Ocean on the Eastern Shore of Virginia. Sites were West Point (37.323, -76.472), York River State Park (37.254, -76.433), Indian Field Creek (37.166, -76.332), Messick Point (37.108, -76.318), and Oyster (37.172, -75.553). Salinity varied along the length of the river at these sites and also seasonally, ranging from 2–35 psu. Squidpops were deployed and scored as described above. At each site we recorded salinity and temperature using a YSI 556–01 Multifunction Water Quality Meter (Yellow Springs Instruments, Yellow Springs OH, USA).

All statistical analyses were conducted using R version 3.1.2. Generalized linear mixed models were fit to a binomial distribution with a log-link using the *lme4* package [32]. For the assays in Belize, the influence of habitat on predation pressure was tested by regressing bait loss against habitat type and scoring time, allowing only the intercept to vary by the random effect of site. In Virginia, the influence of salinity was tested by regressing bait loss against salinity, with month nested within site as a random factor and again allowing only the intercept to vary.

Field deployments at the Goodwin Islands site in Virginia were conducted with permission of the Chesapeake Bay National Estuarine Research Reserve, managed by the Virginia Institute of Marine Science, and at the site near Oyster with permission of the University of Virginia Anheuser-Busch Coastal Research Center. Remaining field deployments in Virginia were conducted immediately adjacent to public boat landings operated by the Virginia Department of Game and Inland Fisheries, where no specific permissions were required. Field deployments in North Carolina were conducted with permission at Hoop Hole (Pole) Creek, a North Carolina Clean Water Preserve managed by the North Carolina Coastal Federation. Deployments in the South Water Caye Marine Reserve, Belize, were permitted by the Belize Fisheries Department. Deployments in Provincia de Bocas del Toro, Panamá, were permitted by the Autoridad de los Recursos Acuáticos de Panamá. Field studies did not involve endangered or protected species. The individuals pictured in this manuscript (Fig 1) have given written informed consent (as outlined in PLOS consent form) to publish these case details.

## Results

### Protocol development

The first protocol test, in Virginia, showed that the composition of the stakes (green plastic-coated metal vs. transparent acrylic) had no significant affect on loss rate of bait. After one hour, the green and transparent stakes had lost 90% and 85% of baits, respectively. In a second run of the same design, green and transparent stakes had lost 25% and 30% of baits, respectively, after one hour, whereas both types had lost 85% of baits after 24 hours.

The second protocol test, in Belize, showed that the length of the tether (1 cm vs. 10 cm) holding the bait did not affect loss rate. After one hour, the sets with short and long tethers had both lost 68% of the baits, and after 24 hours all recovered units (N = 20 short, N = 24 long tether) had lost 100% of the baits. Thus, with respect to tether length and stake composition, we conclude that the squidpop design is relatively robust. Nevertheless, in the interest of minimizing potential confounding factors, we recommend adhering to the standardized design described here to facilitate comparability across studies.

### Predation intensity and predator abundance

**Fig 2:**
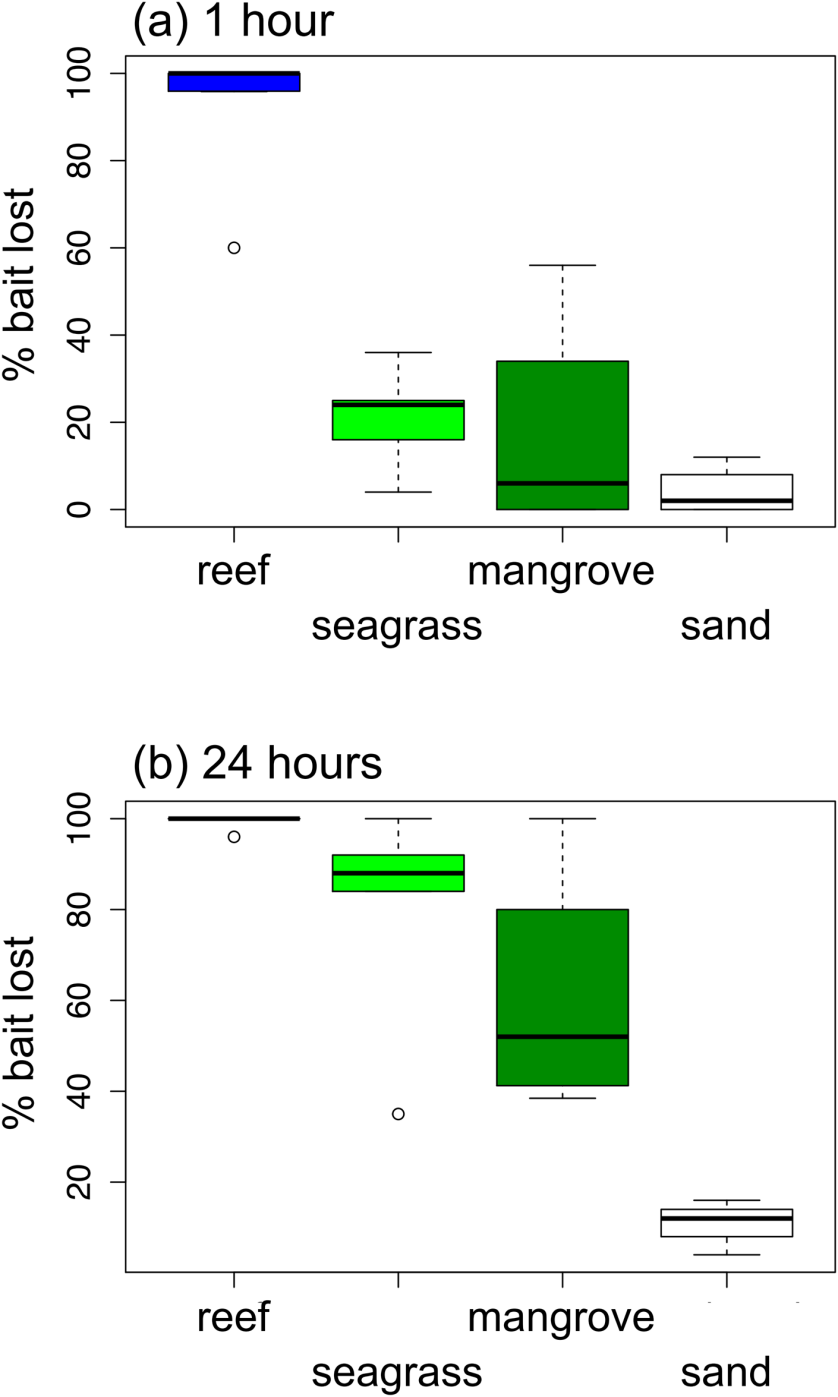
Variation in predation (loss of squid baits) across habitats on the Belize Barrier Reef after (a) one hour and (b) 24 hours. Box and whisker plots show median (thick horizontal line), quartiles (box), 95% percentiles (whiskers) and outliers. Bait loss differed strongly among habitats (P < 0.001), and between 1 and 24 hour scoring times (P < 0.001), with a significant interaction between habitat and time (P = 0.035) based on analysis of deviance from a generalized linear mixed effects model.

Loss of squid baits to predators varied dramatically among habitats in Belize (Fig 2). Nearly all squidpops deployed in the reef habitat were consumed within one hour, whereas fewer than 25% of baits were lost within one hour in seagrass, mangrove, or sand habitats. By 24 hours, however, most baits had also been removed in the seagrass (mean = 80%) and mangrove (61%) habitats, whereas only 11% of baits, on average, were eaten in sand habitats. The results from Belize illustrate the value of scoring bait loss at both one and 24 hours as the 1-hour score separated the reef from all other habitats, whereas the 24-hour score separated sand from all other habitats (Fig 2). Bait loss differed strongly among habitats (P < 0.001), between 1 and 24 hour scoring times (P < 0.001), and there was a significant interaction between habitat and time (P = 0.035).

Squidpop baits were attacked by several species of fishes in Belize. On the reef most observed predation events were by yellowhead wrasse (*Halichoeres garnoti*), but baits were also taken by bluehead wrasse (*Thalassoma bifasciatum*), slippery dick (*Halichoeres bivittatus*), white grunt (*Haemulon plumierii*), redband parrotfish (*Sparisoma aurofrenatum*), and dusky damselfish (*Stegastes adustus*). In the mangroves, observed predation events were by gray snapper (*Lutjanus griseus*). In the seagrass, we did not observe baits being removed, but saw attempts by *T*. *bifasciatum*, *H*. *bivittatus*, scrawled cowfish (*Acanthostracion quadricornis*), slender filefish (*Monacanthus tuckeri*), hamlet (*Hypoplectrus* sp.), and schools of small tomtate (*Haemulon aurolineatum*) nipping vigorously at baits.

**Fig 3:**
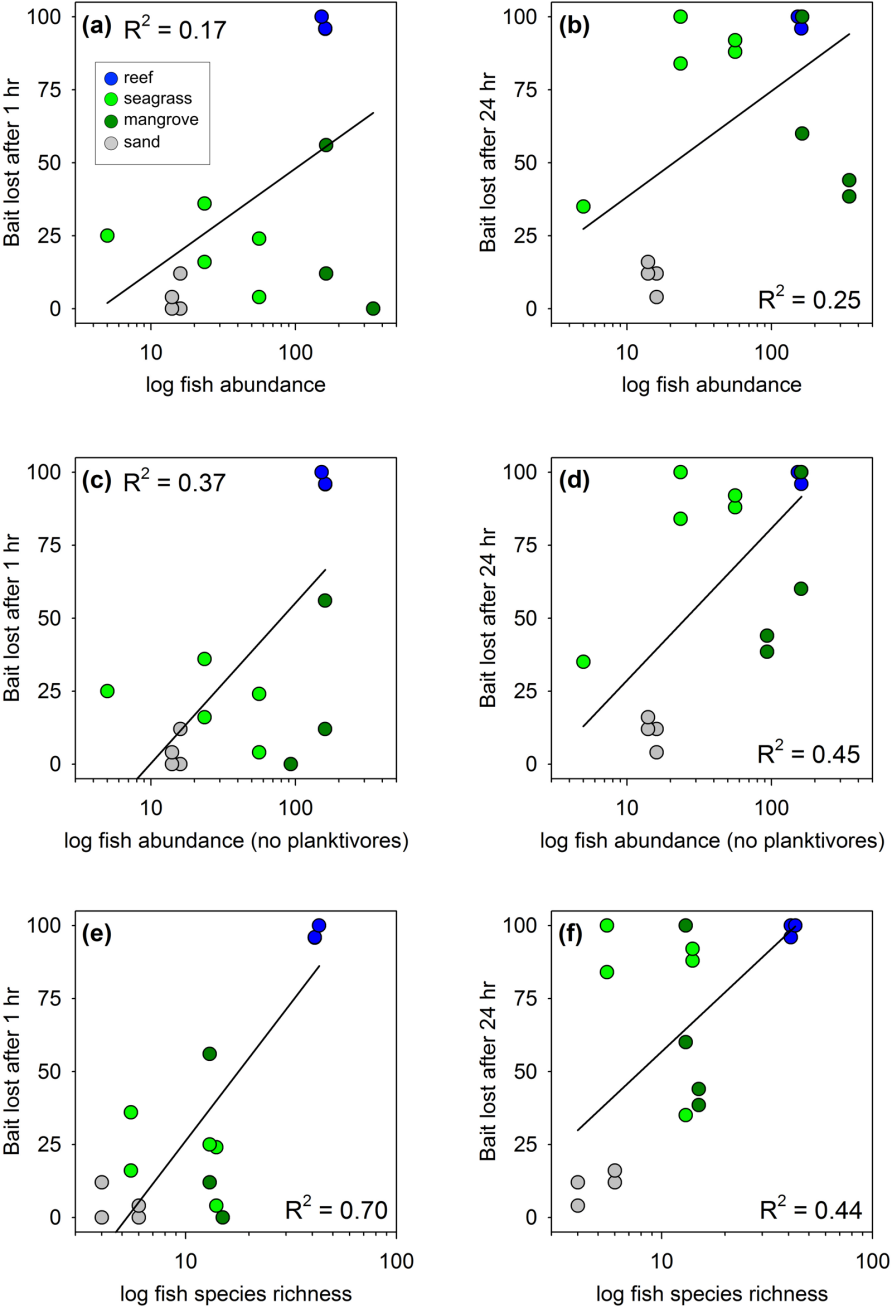
Variation in predation (loss of squid baits) across habitats on the Belize Barrier Reef as a function of fish abundance (a-d) and richness (e,f). Panels b and d show relationships to fish abundance when schooling planktivores are excluded. All relationships are significant (P < 0.05, linear regression).

**Fig 4:**
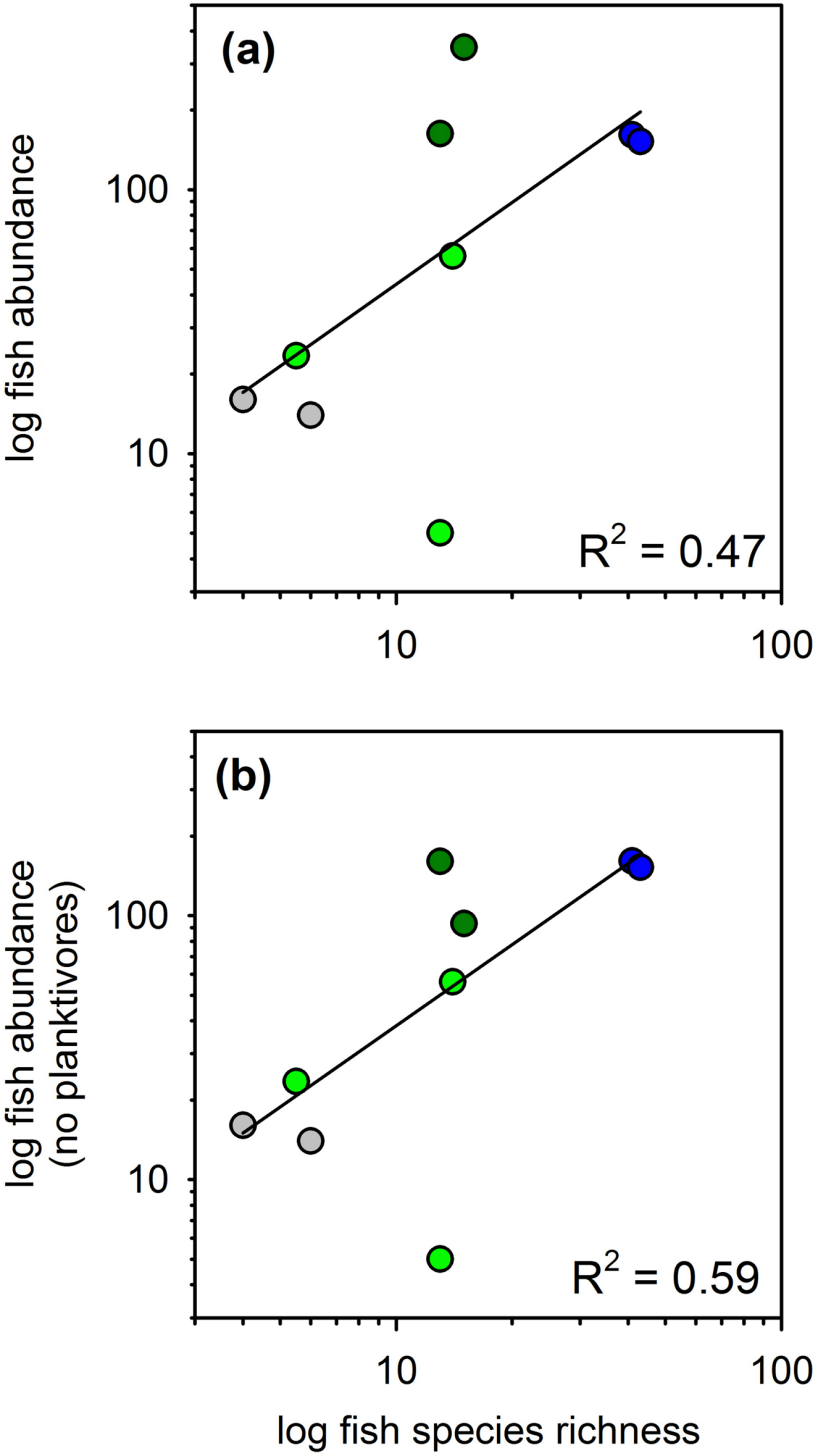
Relationships between fish abundance and richness across habitats on the Belize Barrier Reef. (a) Data for all fishes. (b) Schooling planktivores excluded. Both relationships are significant (P < 0.05, linear regression). Symbol colors as in Fig 3.

Differences across habitats in relative predation intensity, as measured by rate of bait loss, correlated with abundance and species richness of fishes in Belize, but correlations were considerably stronger after excluding planktivorous fishes from the analysis (Fig 3, P < 0.05 for all relationships shown). Some of the mangrove creeks where assays were conducted were frequented by large schools (100s of individuals) of clupeid fishes, including the dwarf round herring *Jenkinsia lamprotaenia* and a larger herring (probably *Odontognathus compressus*). These fishes feed primarily on small prey such as zooplankton in the water column, so were not expected to take baits from the squidpops. When these planktivores were excluded from the analysis, fish abundance explained 37% of the variation in bait loss among deployments at 1 hour, and 45% of the variation at 24 hours (Fig 3). Fish species richness was an especially strong predictor of bait loss at one hour, explaining 70% of the variation, but was comparable to fish abundance at 24 hours (Fig 3). It is unclear whether fish species richness or aggregate abundance is the more important predictor of predation rate since these two are correlated (r^2^ = 0.47), especially when planktivores are excluded (r^2^ = 0.59, Fig 4).

### Variation in predation across seasons and latitude

**Fig 5:**
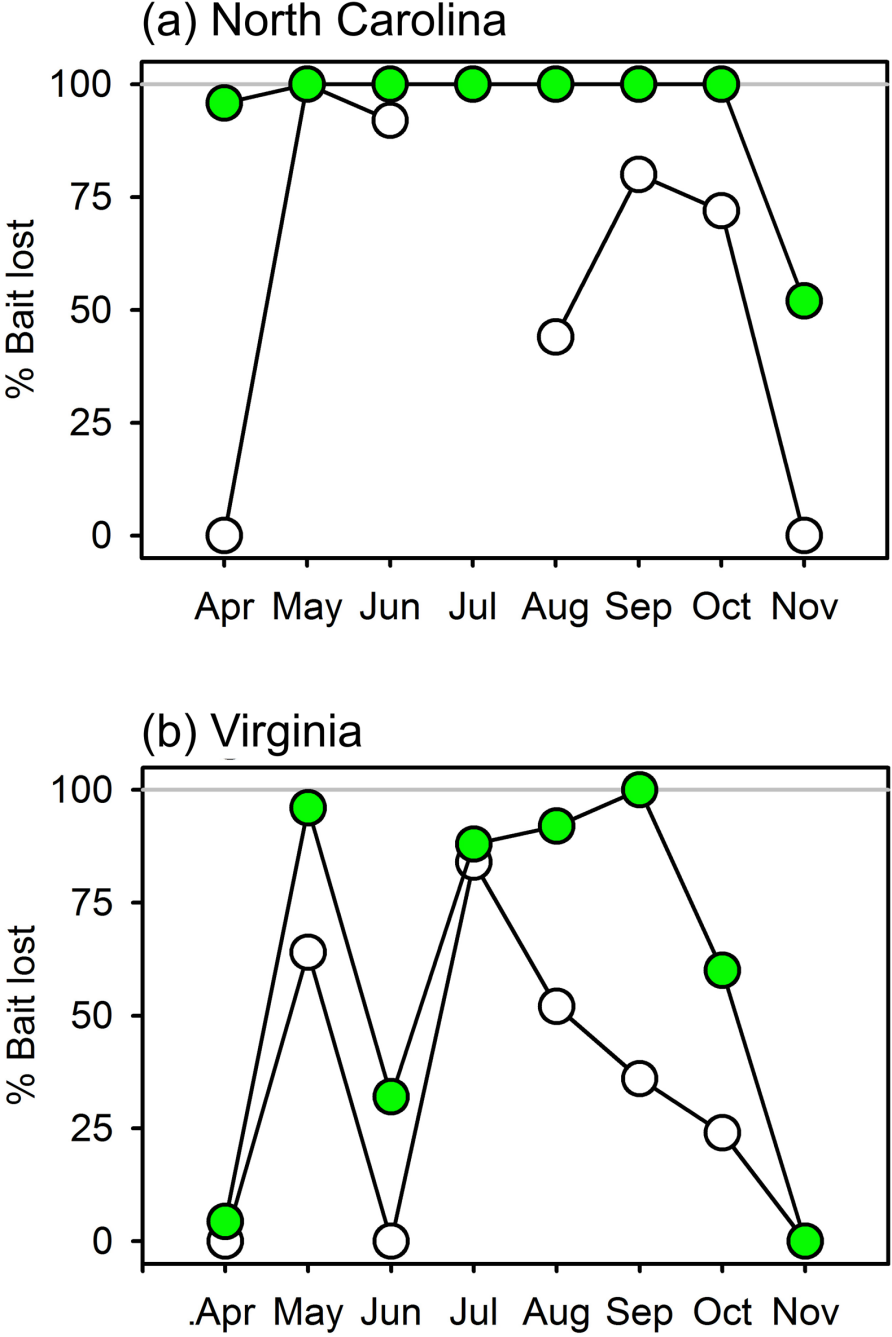
Variation in predation (loss of squid baits) in seagrass habitat across seasons and latitude. Plotted are % of baits lost after 1 and 24 hours at a northern (Virginia) and a southern site (North Carolina), deployed monthly from April through November 2014. Unfilled and filled symbols represent losses at 1 and 24 hours, respectively.

Squidpops deployed monthly in eelgrass beds in Virginia and North Carolina showed that predation intensity varied with season and latitude, being highest at the warmer southern site and in the warmer summer months at both sites (Fig 5). At the more temperate site in Virginia, 24-hour bait loss was negligible during the cooler spring (April) and late fall months (November), and was higher in summer. At the warmer site in North Carolina, bait loss was consistently high (at or near 100%) throughout the period of deployment, only declining to ~50% in November. The results for 1-hour scores were more variable (and not collected in July in NC due to a storm), possibly reflecting tidal variation in the abundance and activities of predators in these shallow systems (<1 m at low tide).

**Fig 6:**
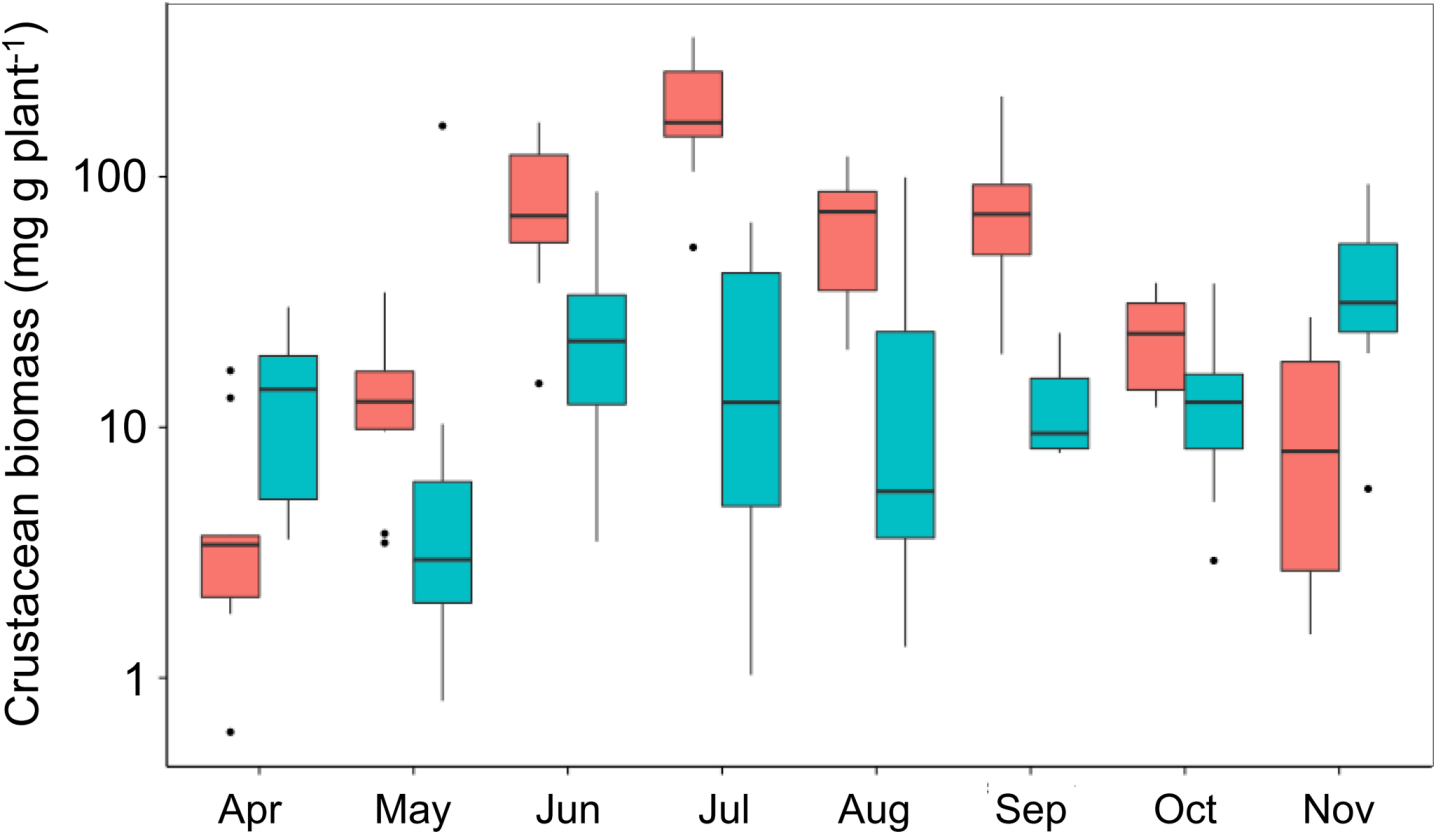
Variation in biomass of epifaunal crustaceans in seagrass habitat across seasons and latitude. Blue and red bars show results from North Carolina and Virginia, respectively, during monthly squidpop deployments between April and November 2014.

Samples of seagrass epifaunal crustaceans collected at each deployment showed more obvious seasonal variation at the northern site in Virginia than at the more southerly site in North Carolina (Fig 6). In Virginia, the estimated biomass of epifaunal crustaceans roughly tracked seasonal temperature trends, rising through the spring to plateau in the summer months (June through September), then declining through October and November. During the summer months crustacean biomass was lower at the North Carolina site, where squidpops revealed generally stronger predation, than at the Virginia site (Fig 5). Thus, within a site epifaunal abundance did not track predation intensity inversely, as would be expected under top-down control, but between the two sites epifaunal abundance was indeed lower at the site with higher predation intensity.

### Variation in predation along a salinity gradient

**Fig 7:**
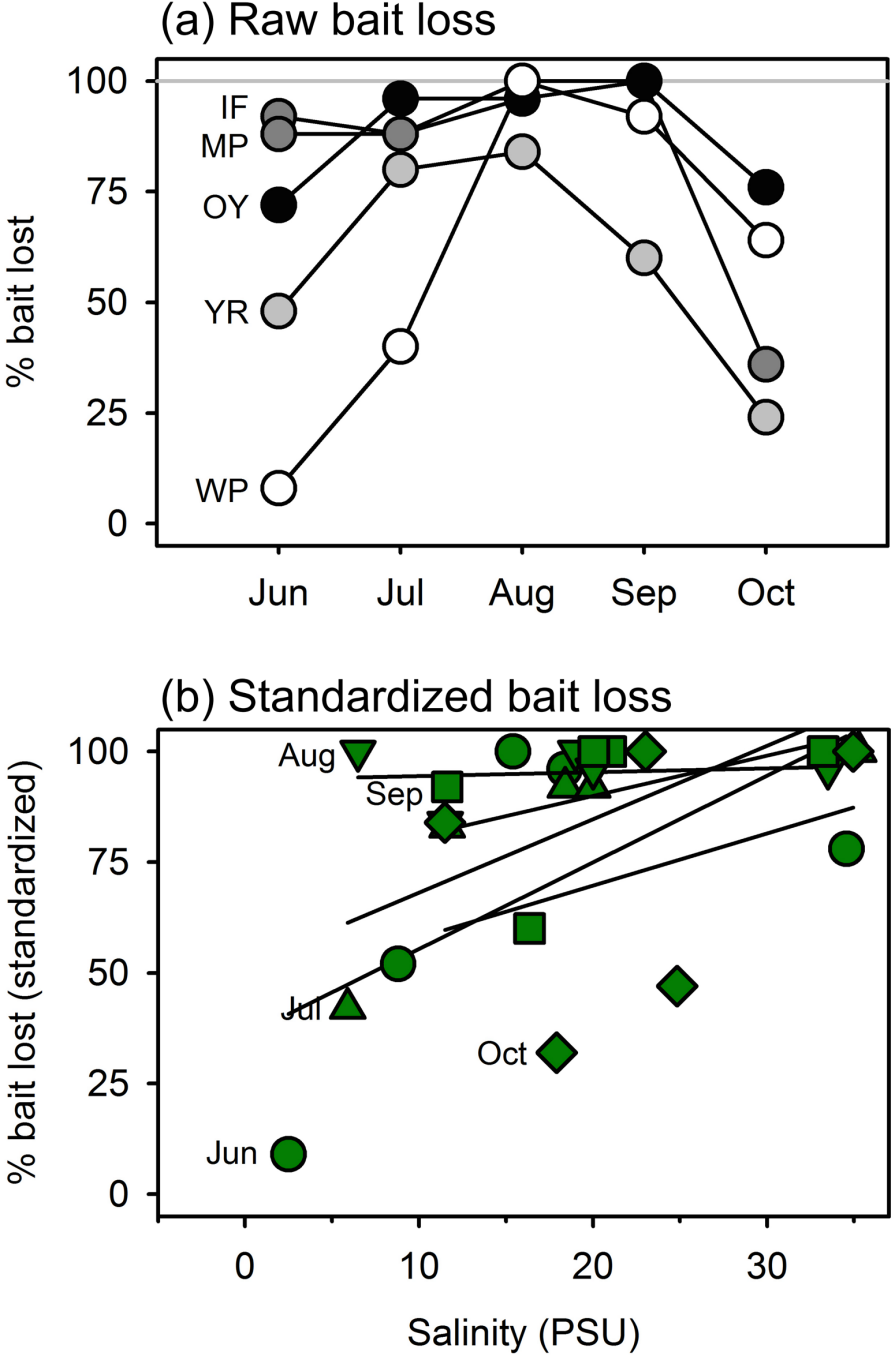
Variation in predation (loss of squid baits) in salt marsh habitat across seasons along a salinity gradient from near freshwater at the head of the York River (West Point, WP) to the ocean margin at Oyster (OY), Virginia, USA, deployed monthly from June through September 2014. (a) Seasonal patterns of bait loss at each of the five sites along the gradient; darker symbols represent higher-salinity sites. (b) Bait loss plotted against salinity at time of deployment. Trend lines are calculated separately for each month, and squid loss data are standardized by dividing by the maximum value recorded during that month. Bait loss increased significantly with salinity (P < 0.001) based on a generalized linear mixed effects model.

In tidal marsh habitats, bait loss increased significantly with salinity, regardless of sampling month (P < 0.001). Loss of squid baits averaged higher and varied less seasonally at the sites with highest salinities compared with those at low salinities (Fig 7a). At West Point, the fresher end of the gradient, predation on baits was near zero in June, rose to near 100% in August, then declined again. At the most saline site (Oyster), in contrast, >70% of baits were lost in every month. Separate comparisons across sites within each month showed generally greater loss of baits with increasing salinity in every month except August, when predation was high at all sites (Fig 7b).

## Discussion

We designed and tested a simple, standardized assay that can rapidly measure the relative intensity of generalist predation in a range of aquatic habitats. Our tests show that the assay captures meaningful variation in predation intensity among habitats, seasons, and along environmental gradients. The observed variation in predation intensity among habitats and seasons matches expectations from theory and prior empirical work that predator activity is higher in warmer temperatures [33], lower latitudes [11], and environments with greater structure [34,35].

Specifically, results in Virginia salt marshes showed that predation intensity decreased upriver into brackish water, consistent with the predator stress hypothesis, which posits that predator activity declines along environmental stress gradients that increasingly challenge organismal physiological tolerances (e.g. osmoregulation) [36]. Results additionally revealed that predation intensity was generally greater in North Carolina than in equivalent habitats farther north in Virginia, consistent with the hypothesis that biotic interactions should weaken with increasing latitude [11]. Predation intensity in Virginia and North Carolina was also greater during summer than in winter, which agrees with both predictions from metabolic theory [33] and documented seasonality of mesopredator abundance [37]. In Belize, predation rates were greatest on structurally complex reefs and lowest in unstructured sand habitats. This pattern presumably reflects the need of mid-sized carnivorous fishes for protection from their own predators, and recalls the halos of grazing seen around patch reefs compared with structureless and ungrazed sand and seagrass beds [38,39].

The dried squid bait was attractive to a wide range of fishes, at least 11 species of which were observed feeding on the baits in Belize, and visual surveys confirmed that variation in predation intensity measured by the assay is well explained by variation among habitats in fish abundance and species richness. Although we were unable to directly observe the predators responsible for taking the squid baits in the turbid waters of Virginia, previous experience suggests that the most likely culprit was the blue crab *Callinectes sapidus*, an abundant generalist predator capable of swimming and thus feeding from squidpops. Therefore, the predators attacking squidpops likely include at least some invertebrates, as well as fishes.

The squidpop assay was designed to be simple, economical, usable in a range of habitats, and relatively foolproof to provide rigorously comparable data across a range of conditions. This simplicity inevitably introduces trade-offs. For example, the assay does not necessarily produce data on absolute rates of predation on actual prey organisms, on feeding selectivity, nor on feeding by mostly benthic invertebrates that cannot reach the tethered bait. Nor is it likely to attract predators attracted primarily by prey behavior. The squid bait is also difficult—but not impossible—for fishes smaller than ~10 cm to remove from the tether, and primarily attracts smaller predators, themselves vulnerable to larger fishes. We believe these limitations are compensated by the assay’s simplicity and standardization. The squidpop assay does what it is designed to do: it provides reliable, standardized data within 24 hours on relative rates of feeding by common generalist predators. That is, the rates of disappearance of bait accurately reflect variation in predator abundance and/or activity among habitats, geographic locations, and time periods. The assay likely measures feeding activity by the most numerous and most active predators in an area, and therefore accurately estimates the relative intensity of aggregate top-down pressure at that site. Whether or not bait loss from squidpops corresponds closely to absolute rates of predation on specific prey organisms by specific predators species requires further study.

The squidpop assay represents a promising tool for characterizing spatial and temporal variation in the intensity of top-down control in aquatic habitats. The assay may prove especially useful for characterizing predation intensity in habitats where it is difficult to census predators accurately, such as in turbid waters with structure that precludes seining. The required materials are inexpensive, readily available, and the assay produces data within 24 hours. The basic protocol is simple but flexible, making it useful in addressing questions across a range of user sophistication. For these reasons, The Smithsonian Institution’s MarineGEO program (http://marinegeo.si.edu/) is implementing squidpops as a standard assay of feeding intensity and has deployed it in several habitat types in Chesapeake Bay, Florida, and Hawaii, USA; Belize; and Caribbean Panama. The *Zostera* Experimental network (www.zenscience.org) is deploying squidpops in eelgrass (*Zostera marina*) habitats at nearly 50 sites throughout the northern hemisphere. The simplicity, economy, standardized design, and rapid results obtainable from the squidpop assay open the door to engaging a wide community of users, including students and citizen scientists, in conducting rigorously comparable measurements to achieve for the first time a high-resolution map, in space and time, of top-down control in aquatic ecosystems.

## Supporting Information

S1 File Data used in the manuscript. (XLSX)
